# Cortical spreading depression as a target for anti-migraine agents

**DOI:** 10.1186/1129-2377-14-62

**Published:** 2013-07-23

**Authors:** Cinzia Costa, Alessandro Tozzi, Innocenzo Rainero, Letizia Maria Cupini, Paolo Calabresi, Cenk Ayata, Paola Sarchielli

**Affiliations:** 1 Neurologic Clinic, Department of Public Health and Medical and Surgical Specialties, University of Perugia, Ospedale Santa Maria della Misericordia, Sant'Andrea delle Fratte, 06132, Perugia, Italy; 2 Neurology II, Department of Neuroscience, University of Torino, Ospedale Molinette, Via Cherasco 15, 10126, Turin, Italy; 3 Fondazione Santa Lucia I.R.C.C.S., Via del Fosso di Fiorano, 00143, Rome, Italy; 4 Neurovascular Research Lab., Department of Radiology, Stroke Service and Neuroscience Intensive Unit Department of Neurology Massachusetts Hospital, Harvard Medical School, 02115, Boston, MA, USA; 5 Neurologic Clinic, Ospedale S. Eugenio, Piazzale Umanesimo, 00144, Rome, Italy

**Keywords:** Cortical spreading depression, Calcium channels, Sodium channels, Glutamate, Ionotropic glutamate receptors, Calcitonin gene-related peptides, Current prophylactic drugs, Antiepileptics, Tonabersat, Gepants

## Abstract

Spreading depression (SD) is a slowly propagating wave of neuronal and glial depolarization lasting a few minutes, that can develop within the cerebral cortex or other brain areas after electrical, mechanical or chemical depolarizing stimulations. Cortical SD (CSD) is considered the neurophysiological correlate of migraine aura. It is characterized by massive increases in both extracellular K^+^ and glutamate, as well as rises in intracellular Na^+^ and Ca^2+^. These ionic shifts produce slow direct current (DC) potential shifts that can be recorded extracellularly. Moreover, CSD is associated with changes in cortical parenchymal blood flow.

CSD has been shown to be a common therapeutic target for currently prescribed migraine prophylactic drugs. Yet, no effects have been observed for the antiepileptic drugs carbamazepine and oxcarbazepine, consistent with their lack of efficacy on migraine. Some molecules of interest for migraine have been tested for their effect on CSD. Specifically, blocking CSD may play an enabling role for novel benzopyran derivative tonabersat in preventing migraine with aura. Additionally, calcitonin gene-related peptide (CGRP) antagonists have been recently reported to inhibit CSD, suggesting the contribution of CGRP receptor activation to the initiation and maintenance of CSD not only at the classic vascular sites, but also at a central neuronal level. Understanding what may be lying behind this contribution, would add further insights into the mechanisms of actions for “gepants”, which may be pivotal for the effectiveness of these drugs as anti-migraine agents.

CSD models are useful tools for testing current and novel prophylactic drugs, providing knowledge on mechanisms of action relevant for migraine.

## Introduction

Spreading depression (SD) is an intense self-propagating wave of depolarization involving neuronal and glial cells in the cerebral cortex, subcortical gray matter or retina, irrespective of functional divisions or arterial territories. This depolarization is followed by a longer lasting wave of inhibition characterised by massive changes in ionic concentrations and slow chemical waves, propagating at a rate of approximately 3–6 mm/min [1]. In both lissencephalic and gyrencephalic cortices, SD can be evoked pharmacologically by the application of K^+^, glutamate, and Na^+^/K^+^-pump inhibitors or by either electrical or mechanical stimulation. SD can develop over the course of epileptic crises or can be induced by brain tissue injury, as in the case of trauma, hemorrhage or ischemia [2-6].

Several clinical and neuroimaging findings support the concept that cortical SD (CSD) is the pathophysiological correlate of the neurological symptoms in migraine aura [7-9]. Moreover, different experimental models of CSD have been developed which help to better understand the underlying neuronal mechanisms and related vascular changes [10,11]. They also, albeit not consistently, suggest that CSD is able to activate central and peripheral trigemino-vascular nociceptive pathways with a latency matching, generally occurring between aura and headache in migraineurs [12].

Familial Hemiplegic Migraine 1 (FHM1) and 2 (FHM2) mutations share the ability to facilitate the induction and propagation of CSD in mouse models, further supporting the role of CSD as a key migraine trigger [13]. CSD is further modulated by endogenous and environmental factors, such as hormones and drugs, and also might be influenced by weather, stress and food [14-16]. Current prophylactic treatments have been investigated for their effects on CSD. Novel drugs can also target CSD and this can account, at least in part, for their mechanisms of action on migraine [17].

## Review

In this review, we illustrate the main findings concerning basic mechanisms underlying CSD as neurophysiologic substrate of aura and report results on the effects on CSD from available drugs and new therapeutic options.

### Clinical, neurophysiological and neuroimaging evidence of a link between migraine aura and CSD

About a third of migraine patients complain of transient focal aura symptoms beginning from minutes to hours before headache, or occurring during either the headache phase or even in its absence [18]. Usually, migraine aura consists of fully reversible visual, sensory and/or dysphasic symptoms [19]. These aura symptoms are accompanied by fully reversible motor weakness in hemiplegic migraine. This is referred to as familial (i.e., FHM) subtype when the condition is present in at least one first- or second-degree relative, and a sporadic subtype in the absence of family history [20].

Specific genetic subtypes of FHM have been identified. Mutations of three genes all encoding ion-channels or membrane ionic pumps were discovered from 1996 to 2005. They involve a neuronal Ca^2+^ channel (CACNA1A, FHM1), a glial Na^+^/K^+^ pump (ATP1A2, FHM2) and a neuronal Na^+^ channel (SCN1A, FHM3), respectively [13]. However, these mutations have not been identified in the more common types of migraine with typical aura. These forms are considered polygenic, with an overall heritability nearing 50%, and should be regarded as the result of the interaction of genetic and environmental factors [21].

Descriptions of migraine with aura (MwA) from the year 1870 onwards have reported a slow, gradual progression of aura symptoms. Specifically, in 1941, Lashley, from the meticulous chartering of his own auras, suggested that aura symptoms reflect a cortical process progressing with a speed of 3 mm/min across the primary visual cortex [22].

CSD was first described by Aristides Leão in 1944 [23,24]. Studying experimental epilepsy for his PhD thesis, Leão came across a depression of electroencephalographic (EEG) activity moving through the rabbit cortex at a rate of 3–6 mm/min after electrical or mechanical stimulations. The negative wave was sometimes preceded by a small, brief positivity, and always followed by a positive overshoot of 3–5 min [25]. He observed that the threshold of CSD varied among cortical areas also in pigeons and cats, and once triggered, it spread in all directions. During CSD, neither sensory stimulation nor direct cortical stimulation evoked potential waves. Ongoing experimental seizure discharge was also suppressed by CSD, even if sometimes tonic-clonic activity preceded or followed SD [23]. Based on similar propagation of the two processes, Leão hypothesized an association between CSD and seizures [1].

Using microscopy and photography of pial vessels to assess cortical circulation, the researcher was also able to see both arteries dilated “as scarlet as the arteries” and veins, as a consequence of CSD. This latter observation, for the first time, indicated that the cerebral blood flow increase exceeded the increase in oxygen demand. This topic has become a matter of interest for all investigators studying changes in cerebral circulation over the course of CSD [24].

In the early 20th century, aura was considered a vascular process involving an initial vasoconstriction followed by a reactive vasodilation responsible for head pain [8,26]. Later observations by Olesen et al. modified this idea by demonstrating a spreading reduction in cerebral blood flow, called “spreading oligemia”, occurring in patients with MwA [27]. This finding completely redefined the underlying pathogenesis of aura by attributing blood flow changes during aura to changes in neuronal activity [28].

Single photon emission computerized tomography (SPECT) [29] and perfusion-weighted magnetic resonance imaging (MRI) [30] studies have further supported the hypothesis that “spreading oligemia” observed during aura is primarily due to changes in neuronal activity. Additionally, perfusion abnormalities have been suggested to be a response of the autoregulatory mechanisms to underlying neuronal depression. However, in most SPECT and hyper-emia and subsequent spreading hypo-perfusion, patients never experienced symptoms of typical visual auras [7,31].

The first-ever study investigating occipital cortex activation during visual stimulation with functional magnetic resonance (fMRI) by blood oxygenation level (BOLD)-dependent contrast imaging in MwA patients demonstrated that the onset of headache or visual change (only in 2 patients), or both, were preceded by a suppression of the initial activation. This suppression slowly propagated into contiguous occipital cortex at a rate ranging from 3 to 6 mm/min. and was accompanied by baseline contrast intensity increases, indicating that vasodilatation and tissue hyper-oxygenation are associated with the induction of headache [32]. Later, in 2001, Hadjikhani at al. [33], strongly suggested that electrophysiological events consistent with CSD are involved in triggering aura in the human visual cortex. Using high-field fMRI with near-continuous recording during visual aura, the authors identified specific BOLD events in the visual cortex that were strictly linked to the aura percept, in both space (retinotopy) and time. Throughout the progression of each aura, unique BOLD perturbations were found in the corresponding regions of the retinotopic visual cortex. Like the progression of the aura in the visual field, the BOLD perturbations progressed from the paracentral to more peripheral eccentricities, in only the hemisphere corresponding to the aura. The source of the aura-related BOLD changes were localized in the extrastriate visual cortex (area V3A) rather than in the striate cortex (V1). Strikingly, the spread rate of the BOLD perturbations across the flattened cortical gray matter was consistent with previous measures of CSD.

As for diffusion changes on MRI, these have been especially observed in cases of prolonged complex migraine aura, suggesting cytotoxic edema in the absence of ischemic lesions [34-37]. To this regard, it is noteworthy the finding of a spreading of cortical edema with reversibly restricted water diffusion from the left occipital to the temporo-parietal cortex in a case of persistent visual migraine aura [38]. In another case of migraine with prolonged aura, hyper-perfusion with vasogenic leakage was detected by diffusion-weighted MRI [39]. A further patient experienced a series of MwA attacks accompanied by slight pleocytosis and gadolinium (Gd-DTPA) enhancement in proximity of the left middle cerebral artery. In this patient, the migraine attacks and Gd-DTPA enhancement were reversed by prophylactic treatment [40].

Magneto-electroencephalography (MEG) allows the study of direct current (DC) neuromagnetic fields in spontaneous and visually induced migraine patients. Few MEG studies have been conducted with this approach in MwA patients due to technical difficulties. The most relevant study to date has shown multiple cortical areas activated in spontaneous and visually induced MwA patients, unlike activation limited to the primary visual cortex in control subjects. This finding supports the hypothesis that a CSD-like neuro-electric event arises spontaneously during migraine aura or can be visually triggered in widespread regions of the hyper-excitable occipital cortex [41].

MEG has also been used to directly register changes in cortical oscillatory power during aura. Specifically, alpha band desynchronization has been demonstrated with this technique in both the left extra-striate and temporal cortex over the period of reported visual disturbances. These terminated abruptly on the disappearance of scintillations, whereas gamma frequency desynchronization in the left temporal lobe continued for 8 to 10 minutes following the reported end of aura [42].

### Neuronal mechanisms of CSD in experimental models

When evoked by local extracellular K^+^ concentrations exceeding a critical threshold, CSD is associated with the disruption of membrane ionic gradients. Both massive K^+^ (with extracellular concentration increases up to 60 mM) and glutamate effluxes are believed to depolarize adjacent neurons to facilitate spread [43]. Results from studies on ion-selective microelectrodes have shown that an extracellular K^+^ rise is accompanied by falls in extracellular Na^+^ and Cl^-^ during CSD, whereas water leaves the extracellular space with significant changes in extracellular pH [44,45].

Specifically, in chick retina, SD induced an initial increase in intracellular pH, which was associated with elevated levels of ADP, P-Creatine, lactate and pyruvate. This was followed by an intermediary acid shift, increases in ATP values and decreases in ADP, a late alkaline rebound, a decrease in P-Creatine levels, and elevations in both ADP and lactate levels. These transient changes in intracellular pH occurring parallel to changes of energy metabolite levels during SD, may be expressions of rapidly modifying metabolic activities of neurons and glial cells. The first alkaline shift was attributed to glial cells, whereas the intermediary acid shift was attributed to neurons. No specific cells were thought to be responsible for the late alkaline shift, which could explain the refractoriness of the neurons in this phase [46]. Accordingly, in rat cerebellum, an initial decrease in [H^+^] (pH increase) followed by a increase in [H^+^] (pH decrease) was observed during SD [45]. Further results obtained from a model of CSD showed acidification and a marked depression in the cortical energy status at the wavefront of SD. Afterwards, a residual activation of glycolysis and an accumulation of cGMP persisted for minutes after relatively rapid restorations of high-energy phosphates and pHi [47]. Recovery from this process occurs usually within a few minutes without any tissue damage [43,45].

A causative link between enhanced glutamate release and facilitation of CSD, induced by brief pulses of high K^+^, has been reported in mouse models of CSD [48-51]. In some of these models, CSD could not be recorded after perfusing cortical slices by Ca^2+^ free medium or after blocking Ca^2+^ channels [50,52-55]. Glutamate involvement in CSD is further supported by the finding of CSD blockage by N-methyl- D-aspartate receptor (NMDA-R) antagonists, but not by non-NMDA-R antagonists, *in vivo*[56-59] or in hippocampal and neocortical slices of rats [5,55]. CSD has also been reported to be blocked by the NMDA-R antagonist 2-amino-5-phosphonovaleric acid, in slices of human neocortical tissue [60]. Furthermore, data have demonstrated that NR2B-containing NMDA-R are key mediators of CSD, providing the theoretical basis for the usefulness of memantine and some NR2B-selective antagonists for the treatment of MwA and other CSD-related disorders, such as stroke or brain injury [50,61].

According to the above findings, CSD cannot be induced in brain slices of FHM1 KI mice if either P/Q-type Ca^2+^ channels or NMDA receptors are blocked. Conversely, blocking N- or R-type Ca^2+^ channels seems to have only small inhibitory effects on the CSD threshold and velocity of propagation. This suggests that Ca^2+^ influx through presynaptic P/Q-type Ca^2+^ channels with consequent release of glutamate from cortical cell synapses and activation of NMDA-R is required for initiation and propagation of the CSD [62]. This is in contrast with results of *in vitro* and *in vivo* pharmacological studies where CSD was induced by perfusing cortical slices with a high K^+^ solution (rather than with brief K^+^ pulses or electrical stimulation). In these models, NMDA-R antagonists only slightly increased CSD threshold without affecting its velocity. Accordingly, blocking P/Q-type (or the N-type) Ca^2+^ did not significantly affect the CSD threshold obtained from perfusing cortical slices with progressively increasing K^+^ concentrations [51,63]. Interestingly, removal of extra-cellular Ca^2+^ did not block CSD but reduced it to about half the rate of propagation [64].

Different results have been obtained for multiple CSD models induced *in vivo* by continuous K^+^ microdialysis or topical application of KCl, where P/Q-type (Cav2.1), or N-type, Ca^2+^ channel blockers and NMDA-R antagonists led to a strongly reduced frequency, amplitude and duration, but not a complete suppression, of CSD events [50,65,66]. Furthermore, Ca^2+^ channel blockers have not been reported to affect CSD induced by pinprick *in vivo*[66]. Therefore, results seem to be strongly influenced by the model used. Currently, electrical stimulation and/or brief applications of high K^+^ are considered to be the most appropriate CSD-inducing stimuli, rather than prolonged applications of high K^+^, for the better understanding of “spontaneous” CSD mechanisms occurring in migraine aura [62]. Specifically, these models best reveal that the excitatory synaptic transmission, involved in CSD initiation and propagation at the pyramidal cortical cells, predominantly depends on presynaptic P/Q-type Ca^2+^ channels.

Earlier studies reported that the Na^+^ channel blocker TTX was not able to consistently inhibit CSD [67-69]. More recently, Na^+^ channels have been shown to be involved in the initiation of CSD in hippocampal slices [5]. Their contribution to CSD was confirmed by Tozzi et al. [70] in rat neocortical slices by reducing CSD propagation after applying the voltage sensitive Na^+^ channel blocker TTX. In another study, Na^+^ ion channel blockage was also seen to inhibit relative cerebral blood flow (rCBF) changes occurring during CSD induced on both cats and rats. In the same model, voltage-dependent Ca^2+^ channel blockers had little effect on either the initiation or propagation of CSD spread, as was the case for ATP-activated K^+^ channel blockers also [71].

It has been demonstrated that the activation of alpha-amino-3-hydroxy-5-methyl-4-isoxazole propionate (AMPA) receptors (AMPA-R) can suppress the actions of NMDA-R in the neocortex [72]. Earlier findings, however, suggested that NMDA-R blockers, but not AMPA-R antagonists, were able to inhibit CSD in rats [70,72,73]. Conversely, a recent study has demonstrated that both 50 μM AMPA, as well as 10 μM of the NMDA-R antagonist 2-amino-5-phosphono-pentanoic acid (2AP5), significantly reduce the number of CSD cycles. Additionally, the gamma-aminobutyric acid (GABA)-mimetic drug clomethiazole (100 mg/kg i.p.) did not significantly affect the number of CSD cycles [74]. Being so, the suppression of NMDA-R actions in the neocortex by AMPA-R activation, may represent an intrinsic protective mechanism against CSD and could, thus, be a potential therapeutic strategy against CSD-related neurological conditions including migraine aura.

In line with the above finding, AMPA-R, as well as GABA(A) and GABA(B)-R agonists, have been shown to inhibit cerebral blood flow changes associated to mechanically-induced CSD in all rats and in a proportion of cats. Furthermore, non-responders showed altered speeds of propagation and times to induction [75]. In contrast, in a recent investigation, reproducible CSD episodes, induced by high extracellular K^+^ concentrations in rat neocortical slices, were inhibited by antagonists of NMDA-R, but not by AMPA-R [70]. Methodological differences (CSD models, dosages of agonists, outcome measures) could explain discrepancy in the results of the different studies carried out on this topic.

Recent autoradiographic findings suggest that selective changes in several receptor-binding sites, in both cortical and subcortical regions, are related to the delayed excitatory phase after CSD. In fact, in neocortical tissues, local increases of ionotropic glutamate receptors NMDA, AMPA, and kainate receptor binding sites have been observed. In addition, receptor binding sites of GABA(A), muscarinic M1 and M2, adrenergic alpha(1) and alpha(2), and serotonergic 5-HT(2) receptors were seen increased in the hippocampus. CSD also up-regulated NMDA, AMPA, kainate, GABA(A), serotonergic 5-HT(2), adrenergic alpha(2) and dopaminergic D1 receptor binding sites in the striatum [76]. Therefore, not only glutamatergic mechanisms, but also changes in monaminergic and cholinergic pathways seem to be involved in CSD.

### Vascular changes associated to CSD in experimental models

CSD has been reported to be associated with changes in the caliber of surface cortical blood vessels.

Leão was the first to report arteriole dilatation accompanying electrophysiological changes in CSD of rabbits [24], which was later confirmed in rats and cats [77,78]. A further study using laser Doppler flowmetry, focusing on tissue perfusion rather than arterial diameter, has suggested that CSD is associated with an initial increase in cortical blood flow, which is thought to correspond to arteriolar dilatation [79]. Triggering CSD results in a sustained wave of reduced cortical blood flow after initial vasodilation, as shown by single modality blood flow measurements, including autoradiographic methods [80,81] and laser Doppler flowmetry [82]. Moreover, sustained hypo-perfusion was accompanied by a concurrent reduction in reactivity to vasoactive stimuli [83]. Dual modality methods, such as laser Doppler flowmetry and extracellular electrophysiology, allowed for the concurrent assessment of changes in neuronal firing and cerebral blood flow in CSD but lacked parallel spatial and temporal resolutions [84]. Optical intrinsic signal (OIS) imaging also enables visualization of CSD on the cortical surface with high temporal and spatial resolution [85-87]. The optical correlates of CSD have been evaluated on both a mouse and a rat model by Ayata et al. [88]. Vascular response to CSD propagates with temporal and spatial characteristics, which are distinct from those of the underlying parenchyma, suggesting a distinct mechanism for vascular conduction.

Using OIS imaging and electrophysiology to simultaneously examine the vascular and parenchymal changes occurring with CSD in anesthetized mice and rats, Brennan et al. [89] observed vasomotor changes in the cortex which travelled at significantly greater velocities compared to neuronal changes. This observation further reinforces the idea that dissociation between vasomotor and neuronal changes during CSD exists. Specifically, dilatation travelled in a circuitous pattern along individual arterioles, indicating specific vascular conduction as opposed to concentric propagation of the parenchymal signal. This should lead to a complete rethinking of flow-metabolism coupling in the course of CSD. Vascular/metabolic uncoupling with CSD has also been reported by Chang et al. using a combination of OIS imaging, electrophysiology, K^+^-sensitive electrodes and spectroscopy in mice [90]. The authors identified two distinct phases of altered neurovascular function. In the first phase of the propagating CSD wave, the DC shift was accompanied by marked arterial constriction and desaturation of cortical hemoglobin. After recovery from the initial CSD depression wave, a second phase was identified where a novel DC shift appeared to be accompanied by arterial constriction and a decrease in tissue oxygen supply, lasting at least an hour. Persistent disruption of neurovascular coupling was supported by a loss of consistency between electrophysiological activity and perfusion.

Nitric oxide (NO) may play a relevant role in determining changes in cerebro-vascular regulation following CSD. In fact, the NO precursor L-arginine prevented the development of prolonged oligemia after CSD but had no influence on a marked rise of CBF during CSD. Moreover, rats treated with L-arginine recovered their vascular reactivity to hyper-capnia after CSD much faster than controls [91]. The NO donor, 2-(N,N-diethylamino)-diazenolate-2-oxide (DEA/NO) had little effect on CSD but reversed the effects of NO synthase (NOS) inhibition by 1 mM L-NAME, in a concentration-dependent manner, suggesting that the increased formation of endogenous NO associated with CSD is critical for subsequent, rapid recovery of cellular ionic homeostasis. Molecular targets for NO may be either brain cells, through the suppression of mechanisms directly involved in CSD or local blood vessels by means of coupling flow with the increased energy demand associated with CSD.

The potent vasoconstrictor endothelin-1 (ET-1) applied on rat neocortices has been demonstrated to induce CSDs through the ET(A) receptor and phospholipase C (PLC) activation. Primary targets of ET-1 mediating CSD seem to be either neurons or vascular smooth muscle cells [92]. This finding provides a bridge between the vascular and the neuronal theories of migraine aura. However, the micro area of selective neuronal necrosis, induced by ET-1 application suggests a role by vasoconstriction/ischemia mechanisms. This observation contrasts with the lack of neuronal damage in several CSD models [93].

### Genetic evidence of CSD involvement in migraine

Genetic factors are known to enhance susceptibility to CSD, as results from transgenic mice expressing mutations associated with FHM or cerebral autosomal dominant arteriopathy with subcortical infarcts and leuko-encephalopathy (CADASIL) have shown [94-99]. Specifically, P/Q-type Ca^2+^ channels, located in somato-dendritic membranes and presynaptic terminals in the brain, play a pivotal role in inducing potential-evoked neurotransmitter release at CNS synapses [100]. Missense mutations in the gene encoding the pore-forming α1 subunit of voltage-gated P/Q-type Ca^2+^ channel, responsible for the rare autosomal dominant subtype of MwA FHM1, induce a gain-of-function of human recombinant P/Q-type Ca^2+^ channels, due to a shift to channel activation at lower voltages [101]. Increased P/Q-type Ca^2+^ current density in cortical pyramidal cells has been demonstrated in Knock-in (KI) mice carrying FHM1 mutations [101-103]. Furthermore, FHM1 KI mice have shown a reduced threshold for CSD induction and an increased velocity of CSD propagation [63,104]. These mice represent a powerful tool for exploring presynaptic regulation associated with expression of P/Q-type Ca^2+^ channels. Mutated P/Q-type Ca^2+^ channels activate at more hyper-polarizing potentials and lead to a gain-of-function in synaptic transmission. This gain-of-function might be responsible for alterations in the excitatory/inhibitory balance of synaptic transmission, favoring a persistent state of hyper-excitability in cortical neurons which may increase the susceptibility for CSD [101]. In contrast, spontaneous CACNA1a mouse carrying mutations producing partial loss-of-function of the P/Q-type Ca^2+^ channel, need approximately a 10 fold higher electrical stimulation intensity in order to evoke a CSD compared to wild-type mice [105].

FHM2, the autosomal dominant form of MwA, is caused by mutations of the α2-subunit of the Na^+^,K^+^-ATPase, an isoform almost exclusively expressed in astrocytes in the adult brain. In a FHM2 KI mouse model carrying the human W887R mutation in the Atp1a2 orthologous gene, *in vivo* analysis of CSD in heterozygous F Atp1a2 (+/R887) mutants revealed a decreased induction threshold and an increased velocity of propagation. While several lines of evidence suggest a specific role on the part of glial α2 Na^+^/K^+^ pump in active reuptake of glutamate from the synaptic cleft, it is plausible that CSD facilitation in the FHM2 mouse model is sustained by inefficient glutamate clearance by astrocytes, leading to an increase in cortical excitatory neurotransmission [106].

MwA is often the first manifestation of cerebral autosomal dominant arteriopathy with subcortical infarcts and leukoencephalopathy (CADASIL), caused by NOTCH3 gene mutations expressed predominantly in vascular smooth muscles. In a recent study, CSD was reported to be enhanced in mice expressing either a vascular Notch 3 CADASIL mutation (R90C) or a Notch 3 knock-out mutation. These findings further support the role of the trigeminal neurovascular unit in the development of migraine aura [107].

### Influence of sexual steroids on CSD

A relation between migraine and changes in the level of sexual steroids has been well documented and both estrogens and androgens may influence migraine attacks. Accordingly, it has been found that in women with MwA, plasma estrogen concentrations were higher during normal menstrual cycle. Furthermore, it has also been reported that the occurrence of migraine attacks is associated with high circulating estrogen levels as during ovulation, pregnancy and the use of certain oral contraceptives [18-110]. Notably, sex difference in the presentation of attacks has been shown to disappear after oophorectomy and with senescence [111]. Testosterone and its synthetic derivatives have also been demonstrated to improve migraine in both men and women [112-116]. Moreover, males treated with gonadotropins for infertility experienced a marked improvement in their MwA attacks [117]. Conversely, anti-androgen therapy increased MwA frequency in a small cohort of male-to-female transsexuals [118].

Some experimental findings support the excitatory neuronal effect associated with estradiol and the inhibitory effect associated with progesterone. Compared to female hormones, mechanisms of androgenic modulation of excitability are not as well known. Gonadic hormones have been suggested to have a modulating role in CSD susceptibility, which would, at least in part, explain the gender differences in the prevalence of migraine. Accordingly, female FHM1 mutant mice have been shown to be more susceptible to CSD when compared to their male counterparts [119]. On the other hand, testosterone have been reported to suppress CSD via androgen receptor-dependent mechanisms and, accordingly, its inhibitory effect on CSD was prevented by the androgen receptor blocker flutamide. Furthermore, it has been shown that chronic testosterone replacement reversed the effects of orchiectomy on CSD [120].

### Astrocytes and gap-junction involvement in CSD

Astrocytes, a subset of glial cells, reside next to neurons, establishing together a highly interactive network [121]. Astrocytes play a pivotal role in limiting CSD by acting as a buffer for the ionic and neurochemical changes which initiate and propagate CSD [122]. On the other hand, astrocyte interconnections are believed to contribute to propagating the CSD wave, by way of K^+^ liberation, allowed for by an opening of remote K^+^ channels. Moreover, energy failure in astrocytes increases the vulnerability of neurons to CSD [123]. There is increasing evidence suggesting that, while synapses connect neuronal networks, gap-junctions most likely connect astrocyte networks [124]. Clusters of these tightly packed intercellular channels allow for the direct biochemical and electrical communications among astrocytes, contributing to a syncytium-like organization of these cells [125]. Membranes of adjacent astrocytes have connexin-containing hemi-channels which can bridge an intercellular gap to form a gap-junction [126]. This interaction between the two hemi-channels opens them both, allowing for the intercellular passage of ions and small molecules [127]. Approximately 1.0–1.5 nm in diameter, gap-junctions permit the transport of molecules up to about 1 kDa in size. Astrocytes express at least three different connexins at gap-junctions with regional differences in their distributions.

Experimental studies have suggested an involvement of gap-junctions in CSD by regulating the *milieu* around active neurons including extracellular K^+^, pH and neurotransmitter levels (especially glutamate and GABA), as well as propagating intercellular Ca^2+^ waves [128]. Non-junctional connexin hemi-channels may also contribute to the release of adenosine triphosphate (ATP). This extracellular messenger is able to mediate Ca^2+^ wave propagation directly or via the transfer of a messenger which triggers ATP release from one cell to another [129]. Generation and propagation of CSD may depend on neuronal activation and Ca^2+^ influx triggered by NMDA-R. Interestingly, NMDA-R antagonists block CSD but, unlike the gap-junction blockers, do not inhibit Ca^2+^ wave propagation.

Astrocytes are known to express several types of glutamate receptors, including NMDA-R. Glutamate release from astrocytes has also have been reported to be mediated via the opening of connexin hemi-channels [127]. For this, gap-junction-mediated propagation of Ca^2+^ waves may represent the advancing front of CSD, contributing to the triggering of the secondary depolarization of the surrounding neurons, leading to further releases of K^+^ and glutamate into the extracellular space. Glutamate may then stimulate cytosolic Ca^2+^ oscillations in astrocytes, providing a feedback loop involved in CSD propagation. If so, gap-junction blockage would represent a viable pharmacological strategy for MwA prevention. Evidence of a gap-junction coupling Ca^2+^ waves between pia-arachnoid cells and astrocytes has also been reported, suggesting a transfer of Ca^2+^ signals from cells of the cortical parenchyma into the meningeal trigeminal afferents, all of which might mediate the induction of neurovascular changes responsible for migraine headache [130].

### Altered blood–brain barrier (BBB) permeability in CSD

CSD alters blood–brain barrier (BBB) permeability by activating matrix metalloproteases (MMPs) [131]. From 3 to 6 hours, MMP-9 levels increase within the cortex ipsilateral to CSD, reaching a maximum at 24 hours and persisting for at least 48 hours. At 3–24 hours, immunoreactive laminin, endothelial barrier antigen, and *zona occludens*-1 diminish in the ipsilateral cortex, suggesting that CSD altered proteins are critical to the integrity of BBB.

Subclinical infarct-like white matter lesions (WMLs) in the brain of some migraine patients, especially those with aura, have been reported to be consistent with CSD-related BBB disruption. Furthermore, increases in plasma levels of matrix MMPs (especially MMP-9 and MMP-2) in migraine patients, in the headache phase, suggest a potential pathogenic role for MMP elevation in both migraine attacks and WMLs [132-135]. Different circulating MMP profiles in MwA and migraine without aura (MwoA) may reflect pathophysiological differences between these conditions. According to Gupta et al. MMPs are responsible for the loosening of the intercellular tight junctions and the expansion of the extracellular matrix of the BBB, consequent to the sudden increase in cerebral blood flow during migraine attacks [136]. In this condition, WML could result from a transient and discrete breakdown of the BBB following sustained cerebral hyper-perfusion rather than hypo-perfusion.

### The relationship between CSD and headache

Recent electrophysiological data has provided direct evidence that CSD is a powerful endogenous process which can lead to persistent activation of nociceptors innervating the meninges. Regardless of the method of cortical stimulation, CSD in rat visual cortices induces a two-fold increase in meningeal nociceptor firing rates, persisting for 30 min or more. Meningeal nociceptors represent the first-order neurons of the trigemino-vascular system, whose activation is involved in the initiation of migraine headache [137]. CSD waves moving slowly across the cortex can promote the releases of K^+^, arachidonic acid, hydrogen ions, NO and ATP. Critical levels of these substances are thought to cause sensitization and activation of trigeminal neurons in the afferent loop and, in turn, activate second-order neurons in the trigemino-cervical complex. These second-order neurons transmit sensory signals to the brainstem and parasympathetic efferents, the latter projecting from the sphenopalatine ganglion. CSD has been suggested to promote persistent sensitization, thereby provoking the activation of meningeal nociceptors through a mechanism involving local neurogenic inflammation, with contribution of mast cells, macrophages and the release of inflammatory mediators. Local action of such nociceptive mediators increases the responsiveness of meningeal nociceptors. Recent research has provided key experimental data suggesting the role of complex meningeal immuno-vascular interactions leading to an enhancement in meningeal nociceptor responses [137]. CSD also induces increased neuronal activity of central trigemino-vascular neurons in the spinal trigeminal nucleus (C1-2) as measured by single-unit recording. It therefore represents a "nociceptive stimulus" capable of activating both peripheral and central trigemino-vascular neurons underlying the headache phase of MwA [137].

Recent evidence suggests that central trigeminal neurons are activated by CSD. Specifically, an increase in the spontaneous discharge rate, following the induction of CSD by cortical injection of KCl was not reversed through the injection of lignocaine into the trigeminal ganglion 20 min after CSD induction. Lignocaine injection prior to the initiation of CSD also failed to prevent the subsequent development of CSD-induced increases in discharge rates [138]. In these experiments, lignocaine at a dosage of 10 μg (capable of interrupting stimulus-induced responses to either electrical stimulation of the *dura mater* or mechanical stimulation of the craniofacial skin) reduced basal the discharge rate of second-order trigeminovascular neurons. This increased traffic in the second-order neurons induced by CSD, however, was not influenced by the blockage of conduction in first-order neurons which was due to lignocaine injection into trigeminal ganglion after CSD induction by cortical pinprick. A time point of 20 min post-lignocaine injection was chosen because responses to evoked stimulation reached a minimum at this time.

It has been suggested that CSD may produce a rapid sensitization at first sensory neurons which could become “locked-in” and, therefore, would not be influenced by a later reduction in sensory traffic, like that induced by the injection of lignocaine into the trigeminal ganglion [139]. An increase in discharge rate produced by CSD has also been observed when lignocaine is injected into trigeminal ganglion, prior to the induction of CSD. This is further evidence that CSD does not act solely by increasing continuous traffic in primary trigemino-vascular fibers through a peripheral action alone, but rather exerts its effect through a mechanism intrinsic to the CNS. Accordingly, pain in MwA may not always be the result of peripheral sensory stimulation, but may arise via a central mechanism [140].

The principal opposition to this hypothesis is based upon the belief that mediators released as a consequence of CSD induction cannot be sustained in the perivascular space to induce persistent trigeminal sensitization and the subsequent hours-lasting headache because of the *glia limitans* barrier (astrocyte foot processes associated with the parenchymal basal lamina surrounding the brain and spinal cord, regulating the movement of small molecules and cells into the brain parenchyma) and the continuous cerebrospinal fluid (CSF) flow [141]. Additionally, the delay of 20–30 min between aura and headache suggests that a time lag is required for the transduction of algesic signals beyond *glia limitans* via inflammatory mediators. In support to this rebuttal, Karatas et al. demonstrated that intense depolarization and NMDA receptor overactivation due to CSD, opens neuronal Pannexin1 (Panx1) mega-channels [142]. Panx1 activation induces a downstream inflammasome formation involving caspase-1 activation and the sustained release of pro-inflammatory mediators from *glia limitans* such as high-mobility group box 1 (HMGB1) and IL-1β, both of which take part in the initiation of the inflammatory response [143-146]. A subsequent NF-kB translocation was observed inside the cortex, involving astrocytes, forming or abutting *glia limitans*, followed by the activations of both cyclooxygenase (COX)2 and inducible NOS (iNOS). The inhibition of Panx1 channels or HMGB1 resulted in a reversal of this effect.

A CSD-induced neuronal megachannel opening may therefore promote sustained stimulus required for both sensitization and activation of meningeal trigeminal afferents through the maintenance of inflammatory responses which may be involved in the subsequent headache pain.

### The effects of anti-migraine drugs on CSD

#### Symptomatic drugs

There is no evidence that acute anti-migraine drugs affect CSD due to the fact that they are not able to block or reduce aura symptoms. In one of the few studies carried out on this, sumatriptan was reported to decrease the amplitude of NO release but was seen to enhance extracellular superoxide concentrations in both lissencephalic and gyrencephalic cortices during CSD [147]. In another study, the same drug failed to inhibit CSD and CSD-related events [148].

#### Preventive treatment

Currently, numerous drugs are available for the prophylactic treatment of migraine, including: tricyclic antidepressants, beta-blockers, calcium channel blockers and antiepileptics. Many of these have been demonstrated to be effective for both MwA and MwoA, suggesting that multiple targets are involved not only in cortical areas but also in sub-cortical structures [17,149]. Results from studies investigating the effects of currently available or novel drugs in animal models for CSD are reported in Additional file 1: Table S1.

#### Current prophylactic drugs

Kaube and Goadsby were the first to investigate the effectiveness of anti-migraine agents in the prevention of CSD by measuring cortical blood flow with laser Doppler flowmetry and cortical single unit activity in alpha-chloralose-anaesthetised cats [150]. None of the tested drugs, including dihydroergotamine (DHE), acetylsalicylic acid, lignocaine, metoprolol, clonazepam and valproate at a single dose prior to CSD induction, were able to inhibit CSD, reduce the rate of propagation or change the amplitude of the cortical blood flow increase. CSD, on the other hand, was blocked by both the NMDA-R blocker MK-801 and halothane. More recently, diverse prophylactic drugs, which are efficacious for the prophylactic treatment of MwA and MwoA, have been shown to experimentally suppress SD susceptibility. In particular, chronic daily administration of topiramate (TPM), valproate, propranolol, amitriptyline, and methysergide dose-dependently were seen to inhibit CSD frequency by 40 to 80% and increase the cathodal stimulation threshold. Longer treatment durations produced stronger CSD suppression. However, the acute administration of these drugs was ineffective [151]. In a further study, peroral administration of TPM, once-daily for 6 weeks, inhibited KCl-induced CSD frequency and propagation. This effect emerged for high plasma levels of the drug, while low levels were ineffective [152]. According to these results, prophylactic drugs should be administered daily for at least 1 month to up 3–6 months, in order to be effective on CSD. This prolonged treatment is necessary to reduce attack frequency and severity in migraineurs. This is due to the fact that pharmacokinetic factors, which determine the gradual achievement of therapeutic tissue levels, as well as the mechanisms involved in gene expression and ultrastructural changes, require such a time period to be effective. In contrast, Akerman and Goadsby demonstrated that mechanically-induced CSD could be prevented by a single dose of topiramate 30 min after administration [153]. Similarly, Hoffmann observed a suppression of CSD susceptibility within 1 hour after a single intravenous dose of gabapentin [154]. No data are available on the effect of high doses of chronic oral gabapentin treatment.

Lamotrigine, a potent Na^+^ channel blocker and glutamate receptor antagonist, has been demonstrated to affect MwA in open-label studies but not MwoA in controlled trials vs placebo or vs an active drug [155-159]. The drug has also been tested for its effect on CSD. Chronic treatment with this drug appeared to exert a marked suppressive effect on CSD, which is in line with its selective action on the migraine aura [160]. Specifically, lamotrigine was seen to suppress CSD by 37% and 60% at proximal and distal electrodes, respectively. Conversely, valproate had no effect on distal CSD, but reduced and slowed propagation velocity by 32% at the proximal electrodes. In the same study, riboflavin had no significant effect. Furthermore, frontal *Fos* expression was decreased by lamotrigine and valproate, but not by riboflavin. Single dose lamotrigine has never been tested for its specific effects on CSD.

Conflicting results have been obtained regarding the effects of the Ca^2+^ channel blocker flunarizine on CSD events [161-166]. These discrepancies are likely due to the different experimental designs utilized, as well as technical limitations characteristic of earlier studies. Moreover, this drug has never been tested on currently used CSD models.

For many years it has been suggested that magnesium (Mg^2+^) deficiency could be involved in migraine pathogenic mechanisms by increasing neuronal excitability via glutamate receptors. To this regard, several studies have reported low values of Mg^2+^ in serum and blood cells over the interictal periods, as well as reduced Mg^2+^ ionized levels in more than 50% of migraine patients during attacks [167-170]. Low brain Mg^2+^ levels have also been detected in migraineurs by brain phosphorus spectroscopy [171]. Furthermore, in MwA patients, magnesium sulphate administration was seen to significantly relieve pain and alleviate migraine-associated symptoms [172]. Accordingly, systemic administration of Mg^2+^ has been shown to reduce CSD frequency induced by topical KCl in rat neocortices [173].

The predictive values of CSD models, specifically for the efficacy of drugs in migraine prophylaxis, can be further reinforced by negative findings from the testing of molecules demonstrated to be ineffective in migraine. This was the case for oxcarbazepine which failed to suppress CSD susceptibility either acutely after a single dose or after chronic treatment for 5 weeks [174] and carbamazepine tested *in vitro* on a CSD rat model [70]. D-propranolol, which is anecdotally ineffective in migraine, also did not suppress CSD, indicating an enantiomer specificity for its efficacy [151].

#### Novel therapeutic options

The novel benzopyran compound tonabersat is a unique molecule, which has been demonstrated to exert activity as a gap-junction inhibitor in animal studies. It has been suggested to be useful in both acute treatment and prophylaxis of migraine [175]. However, conflicting results have been obtained in two-dose ranging, placebo-controlled trials concerning its ability to relieve attacks [176] and also in the two randomized clinical trials aimed at investigating its effectiveness as a preventive drug [177,178]. This lack of efficacy was attributed to the slow absorption of tonabersat, suggesting that a daily administration of higher dosages should be tested for migraine prophylaxis. Interestingly, in another randomised, double-blind, placebo-controlled crossover trial, 40 mg tonabersat, administrated once daily, had a significant preventive effect on MwA but not MwoA, compared to placebo [179]. These findings concur with results of preclinical studies where the drug was reported to markedly reduce CSD and CSD-associated events, compared to sumatriptan [148]. Additionally, a study using repetitive diffusion weighted MR imaging (DWI) detected *in vivo* CSD modulation for tonabersat and, in part, also for sumatriptan [180]. Another mechanism of action of tonabersat includes its ability to inhibit gap-junction communication between neurons and satellite glial cells in the trigeminal ganglion [181]. This mechanism, together with its suppressive effect on CSD and its good pharmacokinetic profile, render the drug a potential candidate for preventive treatment of MwA.

A recent open-labeled pilot study on the Na^+^/H^+^ exchanger amiloride showed that it was clinically effective by reducing aura and headache symptoms in 4 of 7 patients with intractable MwA. Preclinical findings from the same study suggested that the drug blocks CSD and inhibits trigeminal activation in *in vivo* migraine models, via the acid-sensing ion channel (ASIC)1 mechanism [182]. This mechanism could be a novel therapeutic target for MwA. ASIC3 are expressed in most trigeminal neurons where they mediate proton stimulation of calcitonin gene-related peptide (CGRP) secretion [183]. The stimulatory effects of protons (pH 5.5) on CGRP secretion, due to ASIC3 activation, appear not to be limited to peripheral trigeminal neurons, but may also involve dural trigeminal afferents. Activation of dural trigeminal afferents by acidic pH has been shown to be mediated by ASICs channels (most likely ASIC3) but not TRPV1 [184]. Amiloride, due its nonselective inhibitory effect, might influence the activation of these extracellular proton key sensors in trigeminal neurons, therefore reducing CGRP release at both peripheral and central levels.

CGRP is the main mediator of trigeminal pain signals. This neuropeptide acts at several steps in the cascade, from the trigeminal nerve to the CNS. It is released from trigeminal ganglion neurons, both peripherally at the dura and centrally in the spinal trigeminal nucleus and other sites within the CNS. Specifically, both CGRP and CGRP receptors have been observed in structures implicated in the pathogenesis of migraine including cortex and meninges [185]. Activation of CGRP receptors on terminals of primary afferent neurons facilitates transmitter release on spinal neurons and increases glutamate activation of AMPA receptors. Both effects are mediated by cAMP-dependent CGRP mechanisms. CGRP also regulates glia activity within the spinal cord and this activity contributes to central sensitization [186]. Whal et al. investigated for the effect of the CGRP competitive inhibitor CGRP-(8–37) (10^−7^ M) and NO inhibitor NOLAG (10^−4^ M) on dilation of pial arteries accompanying transient negative DC shift during KCl-induced CSD in cats. The authors reported a 75% inhibition of the CSD-induced dilatation during simultaneous application of both compounds, indicating that the initial dilatation during CSD is mediated, at least in part, by releases of CGRP and NO [187]. The latter two may act as mediators of the coupling between neuronal activity and cerebral blood flow during migraine aura. In a further study, topical administration of 12.8 μM CGRP-(8–37) reduced CSD-induced pial vasodilation in urethane-anesthetized rabbits. In the same experiment, the removal of the receptor antagonist from the brain surface restored CSD-induced dilation, further supporting a pivotal role for CGRP in determining transient arteriolar dilation in the first phases of CSD [188]. The above results argue in favor of a local release of CGRP in the meninges, where this neuropeptide may contribute to sensitizing local sensory neurons. However, a recent investigation on cultured cortical astrocytes has shown that CGRP possesses a modest proinflammatory action, as does satellite glia [189].

CSD does not seem to significantly influence CGRP outflow in jugular blood, but local increases of CGRP in the cortex during CSD cannot be excluded [190]. Furthermore, peripheral injection of CGRP in MwA patients has triggered a typical aura in 28% of patients, and migraine-like attacks without aura in the remaining. If this induction of aura can be confirmed, it will indicate that this neuropeptide has an upstream role in CSD [191]. Recent OIS imaging findings support the release of endogenous CGRP during CSD in rat neocortical slices in a calcium-dependent manner [70]. Additionally, three different CGRP receptor antagonists have shown dose-dependent inhibitory effects on CSD events, suggesting the critical role of CGRP in CSD. If so, antagonists targeting central CGRP receptors could be useful as anti-migraine agents [70].

Presently, gepants, a CGRP antagonist class of molecules, might offer a new non-vasoconstrictive approach in the acute treatment of migraine. Four chemically unrelated CGRP receptor (CGRP-R) antagonists (olcegepant, telcagepant, MK-3207 and BI 44370 TA) have displayed efficacy in the treatment of migraine [192]. They have fewer adverse effects, and act for a longer period than triptans [193]. Their development has been slowed by a liver toxicity when used as preventives. New CGRP-R antagonists, such as BMS-927711 and BI 44370 TA, are currently under study. The latter has shown a dose-dependent effectiveness as an acute anti-migraine drug in a recent trial [194]. It remains to be established if these molecules are effective in antagonizing MwA.

#### Other molecules of interest

Kynurenic acid, a derivative of tryptophan metabolism, is an endogenous NMDA-R antagonist whose cerebral concentrations can be increased by the systemic administration of its precursor L-kynurenine. L-Kynurenine administration suppresses CSD in adult Sprague–Dawley rats, most likely by increasing kynurenic acid levels in the cortex. Females are more sensitive to the suppressive effects of L-kynurenine, further highlighting the role of sex hormones in migraine [195]. Ketamine, is a drug primarily used for the induction and maintenance of general anesthesia (usually in combination with a sedative) and also for sedation in intensive care, analgesia (particularly in emergency medicine), and the treatment of bronchospasm. Like other drugs of the same class, such as tiletamine and phencyclidine, it is also used as a recreational drug.

From a pharmacologic point of view, ketamine is an NMDA-R antagonist which, at high fully anesthetic level doses, binds to μ-opioid receptors type 2, without agonist activity and to sigma receptors in rats [196,197]. The drug also interacts with muscarinic receptors, monoaminergic receptors in descending pain pathways and voltage-gated Ca^2+^ channels.

In earlier experiments, ketamine was shown to block CSD in rats [56,198]. As for MK-801, tolerance to ketamine was observed after repeated injections. That is, there was a gradual decline in their CSD blocking effects, which might have been due to some conformational changes at binding site(s) in NMDA-R [199]. Ketamine has been proposed as a putative treatment option for severe and prolonged aura. The first study on ketamine was carried out on 11 patients with severe, disabling auras resulting from FHM. In five of these, the drug reproducibly reduced the severity and duration of the neurologic deficits, whereas in the remaining 6 patients no benefits were observed [200]. A recent double-blind, randomized, parallel-group, controlled study including patients with prolonged aura, reported that intranasal 25 mg ketamine, but not intranasal 2 mg midazolam, reduced aura severity but not duration [201]. However, ketamine has several side effects, including hallucinations, elevated blood pressure, dissociative anesthesia which strictly limit its use in clinical practice.

Early anecdotal evidence indicated that propofol could have been effective in terminating refractory migraine. This rapidly acting water-insoluble non-barbiturate anesthetic agent has been recently reported to be effective on CSD. Specifically, intraperitoneal propofol hemisuccinate (PHS), a water-soluble prodrug of propofol, administered 15 min prior to KCl–induced CSD on the cortex of mice, decreased the number of CSD deflections at doses of 120 and 200 mg/kg without any effect on CSD amplitude [202]. In contrast, Kudo et al. failed to demonstrate any inhibitory effect of propofol on the frequency of KCl-induced CSD in rats [203]. This result may have been due to the fact that a water-insoluble formulation of propofol widely administered as a anesthetic in clinical setting was used. In above mentioned study by Dhir et al. a water-soluble, non-commercially available prodrug PHS, was tested [202]. Considering these contrasting findings, it should be investigated whether metabolites produced during PHS activation, rather than propofol *per se*, can mediate an inhibitory effect on CSD. Given the varying effects of anesthetics on CSD, future studies will have to follow uniform protocols using only anaesthetics having a minimal impact on CSD in order to guarantee reliability and comparability of the results [204].

TRPV1 receptors play an important role in modulating trigeminal sensory processing and for this have been proposed as potential targets for migraine treatment. The TRPV1 receptor antagonist, A-993610, has been tested in a model of mechanically-induced CSD but it failed to show any effects. This lack of effect should be confirmed for other TRPV1 receptor antagonists in future research [205].

Finally, cannabis has been empirically used for centuries for both symptomatic and prophylactic treatment of different types of headaches including migraine. Recent findings have demonstrated a dose-dependent suppression of CSD amplitude, duration and propagation velocity after Delta9-tetrahydrocannabinol (THC) in rat neocortical slices, which had been antagonized by cannabinoid CB1 agonist, WIN 55,212-2 mesylate but not by cannabinoid CB2 agonist, JWH-133 [206]. These finding suggests that cannabinoids might have inherent therapeutic effects for MwA but their known side effects and risk of dependence must be properly weighed before cannabinoid being considered for treatment.

## Conclusions

**Figure 1:**
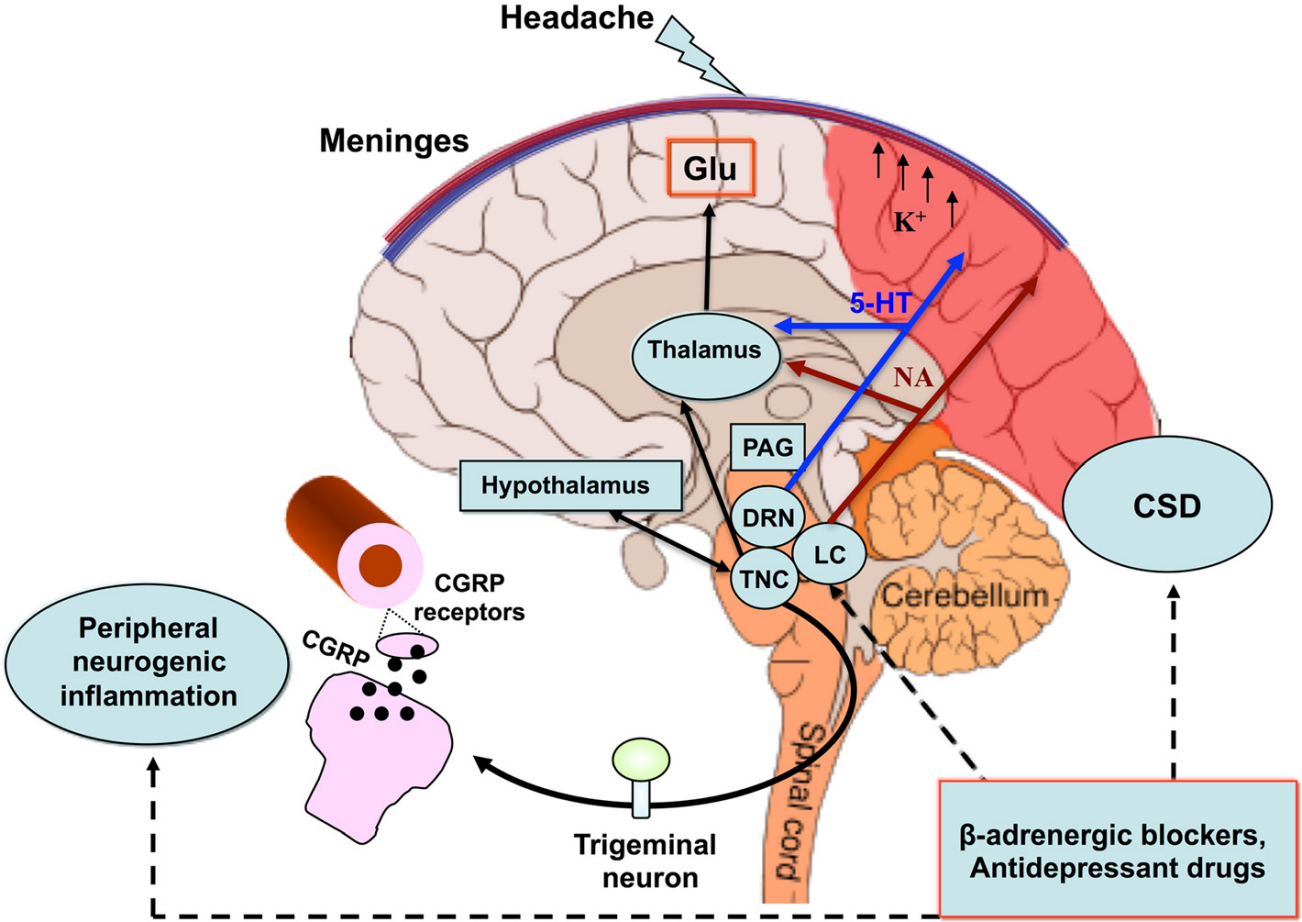
**Mechanisms and structures involved in the pathogenesis of migraine with aura.** CSD underlies aura symptoms. Initiation and propagation of CSD are determined by massive increases in extracellular potassium ion concentration and excitatory glutamate. CSD biochemical changes may trigger the activations of meningeal trigeminal endings and trigemino-vascular system, causing the headache phase. The latter can occur through matrix metalloproteases activation that increases vascular permeability and also through the release of nociceptive molecules from mastcells, including proinflammatory cytokines. The pain phase is due to peripheral and central sensitization of the trigeminal system, as well as to the release of CGRP, both peripherally and centrally. CGRP is considered a key mediator in migraine and, together with NO, is the main molecule responsible for vasodilation consequential to neurogenic inflammation. CGRP is also released from cortical slices during CSD and this calcium-dependent release can mediate the dilatation of cortical arterioles. Periacqueduttal gray matter (PAG), locus coeruleus (LC), nucleus of *raphe magnum* (NRM) are brainstem structures implicated in the processing of trigeminal pain. Functional and structural PAG abnormalities occurring in migraineurs, contribute to the hyper-excitability of trigeminal nociceptive pathways. Functional alteration of noradrenergic nuclei of the LC are believed to be involved in cortical vasomotor instability. Thus, dysfunction in brainstem pain-inhibiting circuitry may explain many facets of the headache phases, even in MwA. CSD participates in this dysregulation by antagonizing the suppressive effect exerted by NRM on the responses of trigeminal neurons. In this scenario, amitriptyline may influence CSD by preserving 5-HT and perhaps NA neurotransmission in the cortex and/or inhibiting high-voltage-activated (HVA) Ca^2+^ channels and Ca^2+^ currents. Propranolol, on the other hand, may reduce neuronal excitability and susceptibility to CSD via a beta-adrenergic blockage. Original brain template was designed by Patrick J. Linch, medical illustrator and C. Carl Jeffe, MD, cardiologist.

A number of mechanisms have been shown to have a role in fostering CSD wave initiation and propagation including: ion diffusion, membrane ionic currents, osmotic effects, spatial buffering, neurotransmitter substances, gap junctions, metabolic pumps, and synaptic connections (Figure 1).

In spite of this knowledge, CSD remains an enigma necessitating further theoretical investigations.

**Figure 2:**
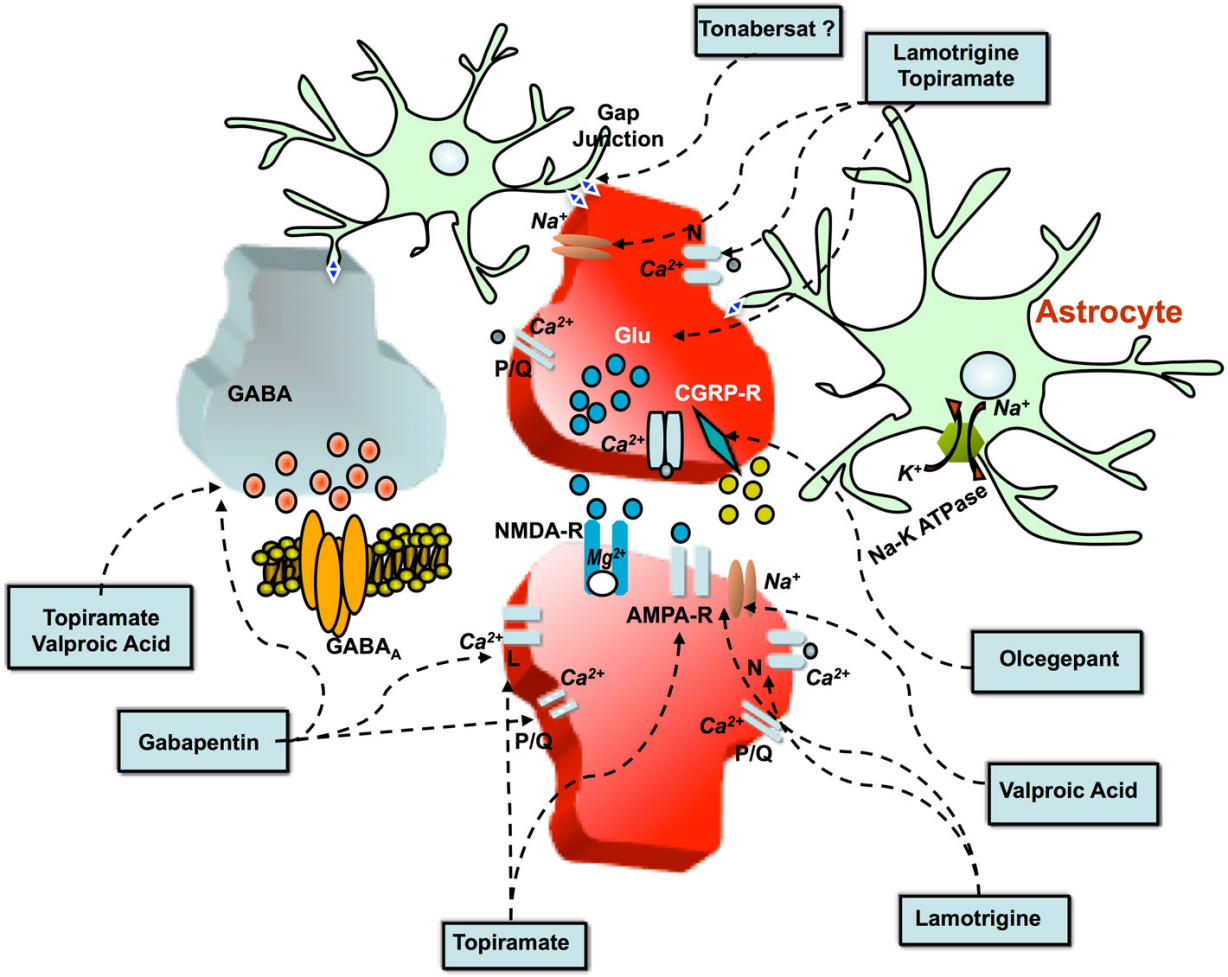
**Main potential targets of currently utilized preventive drugs and those under-investigation for migraine.** The mechanisms mediating CSD inhibition by several migraine preventive drugs are not completely understood. However, it is believed that this inhibitory action is exerted by influencing ion channels and neurotransmission. In particular, topiramate and valproate are believed to exert their antagonizing effects on CSD by blocking Na^+^ channels, inhibiting glutamatergic transmission and increasing GABaergic transmission. Topiramate, but not valproate, can also block L-type Ca^2+^ channels. Lamotrigine has a marked suppressive effect on CSD, which may be due to its selective action on Na^+^ channels. Furthermore, gabapentin is able to modulate the activities of P/Q- and, to a lesser extent, N-type high voltage-activated channels located presynaptically at the cortical level and controls the neurotransmitter release, in particular that of glutamate. This effect and the modulation of GABA-mediated transmission might explain CSD inhibition by gabapentin. Magnesium exerts an inhibitory effect on CSD by interfering with NMDA receptor function and reducing glutamatergic transmission as well as regulating glutamate uptake by astrocytes and modulating NO system in the cortex. The blockage of L-type Ca^2+^ channels may also account for the inhibitory effects of flunarizine on CSD (not shown). The novel benzopyran compound tonabersat seems to inhibit CSD in experimental models and has shown some efficacy in the prophylactic treatment of MwA. It is not known if tonabersat acts on gap-junctions in CNS, as it has already been demonstrated for trigeminal ganglion. CSD inhibition can also be achieved by CGRP-R blockage due to CGRP antagonists, such as olcegepant. This class of drugs might exert its inhibitory effects at both cortical neuronal and cerebrovascular levels.

Experimental findings to date suggest that chronic daily administration of certain migraine prophylactic drugs (topiramate, valproate, propranolol, amitryptiline, and methysergide) dose-dependently suppress CSD. At a molecular level, targets of the inhibitory effects of antiepileptic drugs tested exert their inhibitory effects on CSD targeting Ca^2+^ and Na^2+^ channels, as well as glutamatergic and/or GABAergic transmissions based on their mechanisms of action. Additionally, the antiepileptic drug lamotrigine has a proven suppressive effect on CSD, which could explain its selective action on migraine aura. Preservation of 5-HT, and maybe even NA neurotrasmission in the cortex, could in some way be responsible for the effects of amitryptiline on CSD, while beta-adrenergic blockage by propranolol might facilitate a reduction in cortical neuronal excitability and thus in turn reduce susceptibility to CSD (Figure 2).

Mechanisms of action for some novel molecules are currently under investigation. To date, it has been found that CGRP-R antagonists exert a dose-dependent inhibitory effect on CSD. For this reason, it can be hypothesized that CGRP plays a determining role in CSD and its modulation may be effective for the preventive treatment of MwA (Figure 2). Additionally, tonabersat, a novel benzopyran compound, has been reported to exert an inhibitory action on CSD and on neurogenic inflammation in animal models of migraine. These inhibitory actions might be an effect of the blockage of neuronal-glial cell gap-junctions, as it has only been demonstrated to be for the trigeminal ganglion. The most significant anti-migraine action on the part of tonabersat seems to derive from its inhibitory action on CSD. In fact, clinical trials on tonabersat have shown its preventive effect on MwA attacks but not on MwoA attacks.

Based on recent clinical findings, intranasal ketamine, has been shown to be effective in CSD models, and therefore it has been administered for cases of migraine with prolonged aura, but its use is limited due to its relevant side effects. Noteworthy, the effectiveness of ketamine adds to the existing evidence that glutamatergic transmission plays a role in human aura. The proven capacities of other molecules (i.e. amiloride) in blocking CSD suggests that they might also have a role in preventing MwA.

Further investigations on molecules with evidence of targeting CSD not only would lead to a better understanding of the underlying mechanisms for CSD, but also supply meaningful insight into their potential roles in MwA, as well as other diseases, such as epilepsy, ischemic stroke, intracranial hemorrhage, and trauma, where CSD is thought to be a pathogenic mechanism.

## Abbreviations

5-HT: 5-hydroxytryptamine, serotonin; AMPA: Alpha-amino-3-hydroxy-5-methyl-4-isoxazole propionate; AMPA-R: Alpha-amino-3-hydroxy-5-methyl-4-isoxazole propionate receptor; ATP: Adenosine-5'-triphosphate; ATP1A2: ATPase, Na^+^/K^+^ transporting, alpha 2 (+) polypeptide; BBB: Blood–brain barrier; BOLD: Blood oxygenation level dependent; Ca2+: Calcium; CACNA: Ca^2+^ channel alpha 1A; CADASIL: Cerebral autosomal dominant arteriopathy with subcortical infarcts and leukoencephalopathy; CBFLDF: Cortical blood flow laser Doppler flowmetry; CGRP: Calcitonin gene-related peptide; COX: Cyclooxygenase; CSD: Cortical spreading depression; CSF: Cerebrospinal fluid; DC: Direct current; DEA: (N,N-diethylamino)-diazenolate-2-oxide; DHE: Dihydroergotamine; EEG: Electroencephalographic; ET-1: Endothelin-1; FHM: Familial Hemyplegic migraine; fMRI: Functional magnetic resonance imaging; GABA: Gamma-aminobutyric acid; GABA-R: Gamma-aminobutyric acid receptor; Gd-DTPA: Gadolinium-diethylenetriaminepenta-acetic acid; HMGB1: High-mobility group box 1; IL-1β: Interleukin-1 β; iNOS: Inducible NO synthase; K+: Potassium; KCl: Potassium chloride; KI: Knockin; L-NAME: L-NG-Nitroarginine Methyl Ester; MEG: Magnetoelectroencephalography; Mg2+: Magnesium; MMP(s): Metalloprotease(s); MR: Magnetic resonance; MwA: Migraine with aura; MwoA: Migraine without aura; Na+: Sodium; NA: Noradrenalin; NMDA-R: N-methyl- D-aspartate receptor; NO: Nitric oxide; NOLAG: NO-nitro-L-arginine; NOS: NO synthase; OIS: Optical intrinsic signal; Panx1: Pannexin1; PET: Positron Emission Tomography; PHS: Propofol hemisuccinate; PLC: Phospholipase C; SCN1A: Sodium channel, voltage-gated, type I, alpha subunit; SD: Spreading depression; SPECT: Single-photon emission computed tomography; THC: Tetrahydrocannabinol; TPM: Topiramate; TRPV1: Transient receptor potential cation channel subfamily V member 1; TTX: Tetrodotoxin; WMLs: White matter lesions.

## Competing interests

The authors declare that they have no competing interests.

## Authors’ contribution

All authors equally contributed to the conception and drafting of the manuscript. PC, CA, and PS critically revised the manuscript for important intellectual content. PS, CC, and AT also contributed in the preparation of figures and table. All authors read and approved the final manuscript.

## Supplementary Material

Additional file 1: Table S1 A summary of the most relevant studies on CSD experimental models regarding the effects of currently used drugs and drugs under investigation for migraine prophylaxis. Click here for file
