# Supplementary material for: Cortical spreading depression as a target for anti-migraine agents
Source: J Headache Pain. 2013 Jul 23;14(1):62. doi: 10.1186/1129-2377-14-62 (PMC3728002; doi:10.1186/1129-2377-14-62)
Supplement: Additional file 1: Table S1 — A summary of the most relevant studies on CSD experimental models regarding the effects of currently used drugs and drugs under investigation for migraine prophylaxis. [file 1129-2377-14-62-S1.doc]

**Table 1 A summary of the most relevant studies on CSD experimental models regarding the effects of currently used drugs and drugs under investigation for migraine prophylaxis**

| Drug * | Animals | Model | Administration and dosage (s) | Outcome Measurements | Effects | Comments | Authors [Ref] |
| --- | --- | --- | --- | --- | --- | --- | --- |
| DL-propranolol | Isoflurane-anaesthetised rats | KCl-induced CSD  Electrically-stimulated CSD | Chronic IP injection  20mg/kg/day | Number of CSDs  CSD propagation  Threshold for CSD | Significant dose-dependent reduction  Significant dose-dependent reduction  Significant rise | No effect of D-propranolol  Single doses administered 1 hour before electrical  threshold determination were ineffective | Ayata et al., 2006 [154] |
| Amytriptyline | Isoflurane-anaesthetised rats | KCl-induced CSD  Electrically-stimulated CSD | Chronic IP injection  10 or 20mg/kg/d | Number of CSDs  CSD propagation  Threshold for CSD | Significant dose-dependent reduction  Significant dose-dependent reduction  Significant rise | Single doses administered 1 hour before electrical  threshold determination were ineffective | Ayata et al., 2006 [154] |
| Flunarizine | Alfentanil-anaesthetised rats | KCl-induced CSD | Acute IP. injection  40 mg/Kg | Depression of EEG activity  negative shift in DC potential  threshold for the elicitation of SD | Reversal  Block  Enhancement |  | Reid et al., 1987 [166] |
| Flunarizine | Uretane-anaesthetised rats | KCl-induced CSD | Acute IV injection  1 mg/kg | Cortical hypoperfusion  C fos expression in ipsilateral frontoparietal  Cortex induced by CSD | Inhibition  Significant attenuation | No effect 5-HT2 and histamine H1 antagonist dimetotiazine  Similar effect of the Ca2+ channel blockers, lomerizine (KB-2796) | Shimazawa et al., 1995 [169] |
| Methysergide | Isoflurane-anaesthetised rats | KCl-induced CSD  Electrically-stimulated CSD | Chronic IP injection  0.1 mg and 1mg/kg/  day | Number of CSDs  CSD propagation  Threshold for CSD | Significant dose-dependent reduction  Significant dose-dependent reduction  Significant rise |  | Ayata et al., 2006 [154] |
| Valproate | Alpha-chloralose-anaesthetised cats (females) | Mechanically-induced CSD  (needle prick) | Acute IP injection  60 mg/Kg | Speed of CSD propagation  Cortical blood flow increase during the hyperemic phase | Significant reduction  No change | No effects of DHE, acethylsalicylic acid, metoprolol, clonazepam, lignocaine | Kaube & Goadsby, 1994 [153] |
| Valproate | Isoflurane-anaesthetised rats | KCl-induced CSD  Electrically-stimulated CSD | Chronic IP injection  25, 50, 100, or 200mg/kg/  day | Number of CSDs  CSD propagation  Threshold for CSD | Significant dose-dependent reduction  Significant dose-dependent reduction  Significant rise |  | Ayata et al., 2006 [154] |
| Valproate | Chloral hydrate-anaesthetised rats | KCl-induced CSD | Daily IP  injections for 1 month  200 mg/kg/die | CSD frequency at a distal (parieto-occipital) and a proximal (frontal) electrode  CSD propagation between the two electrode sites  Number of Fos-immunoreactive nuclei in frontal cortex. | Reduced at the anterior recoding site only  Suppressed at the anterior recoding site only by 32% and slowed propagation velocity  Decreased |  | Bogdanov et al., 2011 [163] |

| Topiramate | Alpha-chloralose-anaesthetised rats and cats | Mechanically-induced CSD  (needle prick) | Acute administration by needle plunge into the cortex  30 mg/kg | Cortical depolarization  CSD propagation  regional cerebral hypoperfusion | Inhibited  Inhibited  Inhibited | Inhibition was shown in all rats and 8 out of 13 cats | Akerman & Goadsby, 2005 [154] |
| --- | --- | --- | --- | --- | --- | --- | --- |
| Topiramate |  | KCl-induced CSD  Electrically-stimulated CSD | Chronic IP injection  40, 60, or 80 mg/kg/day | Number of CSDs  CSD propagation  Threshold for CSD | Significant dose-dependently reduced  Long-term suppression  Significantly dose-dependently reduced  Significantly raised |  | Ayata et al., 2006 [154] |
| Topiramate | Rats§ | KCl-induced CSD | In vitro application 10 min after the first episode of CSD  100 μM | CSD area  Intrinsic optical signals (IOS) intensity | Reduced  Reduced | No effect of carbamazepine  Similar effects of  NM DAR antagonist L -A PV) and  Na^+^Channel blocker TTX | Tozzi et al., 2012 [72] |
| Topiramate | Isoflurane-anaesthetised male rats | KCl-induced CSD | Once-daily peroral treatment  50, 100, 200 or 600 mg/kg | Frequency of CSDs  CSD propagation  Interval between CSD episodes | Significantly dose-dependently reduced  Long-term suppression  Significantly dose-dependently reduced  Lengthened |  | Unekawa et al., 2012 [155] |
| Gabapentin | Isoflurane-anaesthetised rats | KCl-induced CSD  Electrically-stimulated CSD | Single IV injection (100 or 200mg/kg) | Frequency of CSDs  Speed of CSD propagation  Threshold for CSD | Dose-dependently reduced the frequency of CSDs by up to 30% within 1 hrs  Not changed  Significantly raised |  | Hoffmann et al., 2010 [157] |
| Lamotrigine | Chloral-hydrate- anaesthetised rats | KCl-induced CSD | Daily I.P. injections for 1 month  15 mg/kg/die | Frequency of CSDs at a distal (parieto-occipital) and a proximal (frontal) electrode  CSD propagation between the two electrode sites  Number of Fos-immunoreactive nuclei in frontal cortex. | Reduced at the proximal and distal electrodes  Suppressed by 37% and 60% at proximal and distal electrodes.  Decreased | No effect of riboflavin | Bogdanov et al., 2011 [163] |
| Magnesium sulphate | Fentanyl citrate and fluanisone- anaesthetised rats | KCl-induced CSD | Acute IV injection  Given. at 90 min after onset of CSDs  10/mg/kg | Frequency of CSDs | Significantly reduced | Similar effect of MK-801 | van der Hel et al., 1998 [176] |
| Novel potential therapeutic options |  |  |  |  |  |  |  |
| Tonabersat | Halotane-anaesthetised rats | KCl-induced CSD | Acute IP injection  10 mg/ kg | Cortical extracellular field potential depolarisations  Cortical and brain stem cGMP by tonabersat | Significantly reduced  Brain stem cGMP was abolished | No effect of sumatriptan | Read et al., 2001 [151] |
| Tonabersat | Halotane anaesthetised cats, subsequently maintained with alpha-chloralose # | KCl-induced CSD | Acute IP and IV injection  10 mg/ kg | Apparent diffusion coefficient (ADC) measured *in vivo* by diffusion weighted images | Significantly reduced ADC | Changes of ADC express reduced of magnitude and number of CSD events initiated and duration of CSD activity .  These effects are partially shared by sumatriptan | Bradley et al., 2001 [183] |
| CGRP antagonists  CGRP 8-37  MK-8825  BIBN4096BS | Isoflurane-anaesthetised rats | KCl-induced CSD | Incubation with solutions of the antagonists of neocortical slices  (2 hrs before CSD induction)  10 μ M  10 μ M  0.1 μ M | CSD area  Intrinsic optical signals (IOS) intensity | Significantly reduced  Significantly reduced | Exogenous CGRP partially reverted the inhibition  evoked by MK-8825 | Tozzi et al., 2012 [72] |
| Other drugs of interest for migraine |  |  |  |  |  |  |  |
| Memantine  [pan-NMDA-R blocker] | Isoflurane- anesthesized rats | KCl-induced CSD | Acute administration  1, 3, and 10 mg/kg | CSD event number  CSD amplitude | dose-dependently decreased  Significantly reduced | Similar results were obtained for two antagonists with selectivity for NMDA-R containing the NR2B subunit (CP-101,606 and Ro 25-6981) | Peeters et al., 2007 [51] |
| Amiloride  [Acid-Sensing Ion Channel 1 (ASIC 1) blocker] | Propofol or isoflurane anesthesized rats (males) | Mechanically-induced CSD  (needle prick)  Electrical stimulation of the thinned cranium | Acute IV injection  10mg/kg | CSD event number  Neurogenic vasodilation of middle meningeal artery  Neuronal response of A-fiber input to dural electrical stimulation | Significantly inhibited  Significantly reduced  Significantly inhibited | The selective ASIC1a blocker psalmotoxin inhibited CSD  CSD was blocked only in 1 of 8 ASIC 1 knockout (ASIC1-/-) mice with deletion of the ACCN2gene | Holland et al., 2012 [185] |

**Legend**

* The order of the drugs presented in the Table has been adopted from current guidelines: beta-blockers, tricyclic antidepressants, Ca^2+^ channel blockers, antiepileptics, magnesium and other potentially useful anti-migraine molecules

§ This was an in vitro study and therefore the effects from the anesthesia (isoflurane) were most likely dissipated at the time of cortical slice testing

# Alpha-chloralose was used to maintain anesthesia due to the known effects of alotane on CSD events

Abbreviations : ADC: Apparent diffusion coefficient; cGMP: cyclic guanosine monophosphate; ASIC 1: Acid-Sensing Ion Channel 1; DHE: Dihydroergotamine; IP: intraperitoneal; IOS: Intrinsic optical signals; IV: intravenous; NMDA-R : N-methyl-D-Aspartate Receptor
